# Case report: MicroRNA-10b as a therapeutic target in feline metastatic mammary carcinoma and its implications for human clinical trials

**DOI:** 10.3389/fonc.2022.959630

**Published:** 2022-10-26

**Authors:** N. Anna Savan, Paulo Vilar Saavedra, Alan Halim, Vilma Yuzbasiyan-Gurkan, Ping Wang, Byunghee Yoo, Matti Kiupel, Lorenzo Sempere, Zdravka Medarova, Anna Moore

**Affiliations:** ^1^ Precision Health Program, Michigan State University, East Lansing, MI, United States; ^2^ Small Animal Clinical Sciences, College of Veterinary Medicine, Michigan State University, East Lansing, MI, United States; ^3^ Microbiology and Molecular Genetics and Small Animal Clinical Sciences, College of Veterinary Medicine, Michigan State University, East Lansing, MI, United States; ^4^ Department of Radiology, College of Human Medicine, Michigan State University, East Lansing, MI, United States; ^5^ Athinoula A. Martinos Center for Biomedical Imaging, Department of Radiology, Massachusetts General Hospital, Charlestown, MA, United States; ^6^ Pathobiology and Diagnostic Investigation, College of Veterinary Medicine, Michigan State University, East Lansing, MI, United States; ^7^ Transcode Therapeutics Inc., Boston, MA, United States

**Keywords:** miRNA, breast cancer, metastasis, large animals, nanoparticles, magnetic resonance imaging

## Abstract

Ninety percent of deaths from cancer are caused by metastasis. miRNAs are critical players in biological processes such as proliferation, metastasis, apoptosis, and self-renewal. We and others have previously demonstrated that miRNA-10b promotes metastatic cell migration and invasion. Importantly, we also showed that miR-10b is a critical driver of metastatic cell viability and proliferation. To treat established metastases by inhibiting miR-10b, we utilized a therapeutic, termed MN-anti-miR10b, composed of anti-miR-10b antagomirs, conjugated to iron oxide nanoparticles, that serve as delivery vehicles to tumor cells *in vivo* and a magnetic resonance imaging (MRI) reporter. In our previous studies using murine models of metastatic breast cancer, we demonstrated the effectiveness of MN-anti-miR10b in preventing and eliminating existing metastases. With an outlook toward clinical translation of our therapeutic, here we report studies in large animals (companion cats) with spontaneous feline mammary carcinoma (FMC). We first investigated the expression and tissue localization of miR-10b in feline tumors and metastases and showed remarkable similarity to these features in humans. Next, in the first case study involving this therapeutic we intravenously dosed an FMC patient with MN-anti-miR10b and demonstrated its delivery to the metastatic lesions using MRI. We also showed the initial safety profile of the therapeutic and demonstrated significant change in miR-10b expression and its target HOXD10 after dosing. Our results provide support for using companion animals for further MN-anti-miR10b development as a therapy and serve as a guide for future clinical trials in human patients.

## 1 Introduction

Breast cancer is the most diagnosed type of cancer in women (1) with the most (death) due to metastasis, wherein cancer cells colonize distant organs (2). Since solutions for patients with metastatic disease are limited, prevention and elimination of breast cancer metastases present an unmet clinical need that must be addressed.

MicroRNAs (miRNAs) are small non-coding RNAs that regulate gene expression (3, 4) and play critical roles in proliferation, metastasis, apoptosis, and self-renewal in various cancers (5–7). In particular, microRNA-10b (miR-10b) has been implicated in invasion and migration in breast cancer and identified as one the drivers of the metastatic process (8–10).

In our earlier studies, we discovered that in addition to promoting invasion and migration of tumor cells, miR-10b serves as a critical driver of metastatic cell viability and supports the survival of these cells outside the primary tumor (11–13). These findings prompted us to develop a strategy for treating metastatic cancer based on miR-10b inhibition. This was achieved using the therapeutic composed of anti-miR-10b antagomirs, conjugated to iron oxide nanoparticles, which serve as delivery vehicles for antagomirs to primary tumors and metastases (MN-anti-miR10b; TTX-MC138 under clinical development) (11, 13, 14). Magnetic properties of these nanoparticles allow for monitoring of their delivery *in vivo* using magnetic resonance imaging (MRI), representing an added value for the clinical implementation of this therapeutic approach. In our previous studies in murine models of metastatic breast cancer, we demonstrated that MN-anti-miR10b combined with low-dose chemotherapy caused complete elimination of local (12) or distant (13) metastases in immunocompromised and immunocompetent models, respectively.

With an outlook toward the clinical translation, our next step was to test the scalability of our therapeutic strategy in large animals with spontaneous feline mammary carcinoma (FMC), which is the third most common cancer in cats (15, 16) and is highly metastatic (17). It has high resemblance to human breast cancer compared to mammary carcinomas of other companion animals in terms of relative age of onset, incidence, risk factors, prognostic aspects, histopathology, biological behavior, metastatic pattern and response to therapy (15–20). Importantly, felines experience the same environmental risk factors as humans and are immunocompetent, more accurately reflecting the complex interplay between genetics, the immune system, and the tumor microenvironment. Finally, there is a greater homology between cats and humans than is between rodents and humans for specific genes (18, 21, 22). For these reasons cats are considered the best large animal model for human breast cancer by most researchers.

Here, we report our initial studies in feline patients, demonstrating similarity of FMC to that in humans in terms of miR-10b expression and histological features in both HER2+ and TNBC types. We also report on the first case of a feline patient (Case 0) with FMC dosed with MN-anti-miR10b that evaluated the potential use of cats with spontaneous tumors for preclinical drug testing. Since the delivery of MN-anti-miR10b to the site of interest is a prerequisite for successful therapeutic intervention, we first investigated its accumulation in Case 0 patient using *in vivo* MRI and explored the initial safety profile of the therapeutic. We also evaluated target engagement of the therapeutic judged by the change in the expression of miR-10b and its target, Homeobox D10 (HOXD10), after dosing. Our results provide compelling evidence for the potential of MN-anti-miR10b as a therapy for metastatic mammary carcinoma in companion animals and serve as a guide for future clinical trials in human breast cancer patients.

## 2 Materials, methods and case description

### 2.1 Tissue collection and expression analysis

Archival blocks of formalin-fixed paraffin-embedded (FFPE) tissue (matched primary tumors and metastatic lymph nodes) from companion cats diagnosed with mammary carcinoma (n=9, 44%TNBC, 56%HER2+) were obtained from the tissue bank of the Michigan State University (MSU) Veterinary Diagnostic Laboratory (VDL).

RNA was isolated using the RecoverAll™ Total Nucleic Acid Isolation Kit for FFPE (Invitrogen, AM1975), and reverse transcription was performed using the miScript II RT Kit (Qiagen, 218161) according to the manufacturer’s instructions. The miScript SYBR Green PCR Kit (Qiagen, 218076) was used for qPCR reaction with the appropriate specific forward primer for miR-10b: 5’-TACCCTGTAGAACCGAATTTGTG-3’ and snRNAU6: 5’-GCAAGGATGACACGCAAATTC-3’ in combination with the universal reverse primer provided in the kit. Forward and reverse primer set for HOXD10 was F1 5’-ATTGTCCTTGGTGAGATGGAAT-3’ and R1 5'-GGACAGGTTGCTGTTGTACT - 3'. MicroRNA expression levels were detected on a CFX96 Touch Real-Time PCR Detection System and data were analyzed using CFX Maestro software (Bio-Rad Laboratories, Hercules, CA). miRNA-10b expression in biopsy and necropsy samples from Case 0 was analyzed using qRT-PCR as described above.

### 2.2 Tissue staining and image analysis

Staining was conducted using FFPE sections for HOXD10 staining, deparaffinization, antigen retrieval, immunohistochemical labeling, and counterstaining (BOND-MAX automated staining system, Leica BioSystems, Buffalo Grove, Illinois). Following CC1 retrieval (Leica BioSystems), slides were labeled with an anti-HOXD10 antibody (LS-B14548, Lsbio), and labeling was detected with the BOND Polymer Refine Detection kit (Leica Microsystems) and visualized with a diaminobenzidine (DAB) detection system and hematoxylin counterstaining. Multiplex fluorescence-based *in situ* hybridization (ISH) probing miR-10b and HOXD10 expression was performed using a Leica Bond Rx automated staining station (23).

Immunohistochemistry (IHC) for HER2+, ER, and PR was performed as previously described (16). For histopathology, tissues were stained with hematoxylin and eosin (H&E) and acquired and analyzed using an Aperio Versa 8 Brightfield & Fluorescence imaging system (Leica Biosystems, Buffalo Grove, IL) with customized narrow-width band excitation and emission filter cubes (Chroma Technology Corp., Bellows Falls, VT). The ImageScope tools were used for annotation and quantitative analysis.

To confirm accumulation of MN-anti-miR10b in biopsy and necropsy samples the obtained tissues were counterstained with Vectashield mounting medium containing 4’,6-diamidino-2-phenylindole (DAPI; Vector Laboratories). Images were acquired using a Nikon Eclipse 50i fluorescence microscope, analyzed using ImageJ software (NIH, Bethesda, MD), and converted to grayscale or RGB stacks. Threshold correction was applied, and histograms were generated for each image.

### 2.3 Case description in a patient with FMC (Case 0)

#### 2.3.1 Synthesis of MN-anti-miR10b therapeutic

Aminated dextran-coated iron oxide superparamagnetic nanoparticles were synthesized and labeled with the near-infrared optical Cy5.5 dye according to a protocol published by us previously (12, 13). Nanoparticles with a size of 20.3 ± 0.6 nm were used for conjugation to the oligonucleotides. The LNA antagomirs (Biospring, Frankfurt, Germany) were synthesized with the 5’-thiol-modifier C6 disulfide (5’-ThioMC6) for conjugation to dextran-coated iron oxide nanoparticles (5’-ThioMC6-D/TCGGTTCTACAGGGT-3’). The nanoparticles were first conjugated to the heterobifunctional crosslinker N-succinimidyl 3-[2-pyridyldithio]-propionate (SPDP; Thermo Scientific Co., Rockford, IL) and then to the activated oligos. The 5’-ThioMC6 of the oligo was activated to release thiol *via* 3% TCEP treatment in nuclease-free PBS and purified using an ammonium acetate/ethanol precipitation method. The number of oligos per nanoparticle was 8.0 ± 0.7, as determined by electrophoresis as described previously (12, 13). Prior to injection, the nanoparticle solution was sterilized by passing through a 0.22µm filter (Thermo Fisher).

#### 2.3.2 Studies with the feline mammary carcinoma patient

A fourteen-year old spayed feline patient (Domestic Shorthair cat, 2.9kg) diagnosed with metastatic mammary carcinoma in the left third mammary gland and treated at the Medical Oncology Service of the College of Veterinary Medicine at MSU was the subject of this study. Prior to enrollment, the patient underwent radical mastectomy of the entire left mammary chain and lymph nodes followed by chemotherapy with doxorubicin, carboplatin, lomustine and cyclophosphamide. Subsequently, the cat developed secondary tumors/metastatic lesions in the subcutaneous ventral abdomen along the linea alba and ventral iliac area confirmed by computed tomography (CT) (GE Revolution EVO 64 Slice CT scanner) and was scheduled for euthanasia in mid-May due to overall poor health and significant weight loss. After receiving IRB and IACUC approvals and the owner’s consent, on June 27^th^ the patient was subjected to MR imaging before and 24 h after intravenous injection of Cy5.5-labeled MN-anti-miR10b (2.5mg iron/kg; 1.4mg oligo/kg). MRI was performed at a clinical 1.5T scanner (General Electric) using T2W sequences (TR/TE 3960.00/108:00ms, slice thickness: 3.5mm) and T2* maps (TR/TE405.00/5.10ms, 14.06ms, 23.02ms, 31.98ms, 41.41ms, slice thickness: 3.5mm). Biopsy of the metastatic lesions was performed immediately after the imaging session.

At the request of the owner, the second dose of MN-anti-miR10b (2.5 mg iron/kg; 1.4 mg oligo/kg) was approved by the medical oncology team and administered seven weeks after the first dose on August 17th. This animal survived for an additional 3 months (13 weeks), after which it was euthanized on November 16th due to disease progression and a decrease in quality of life. Tumor samples at necropsy were processed for histology as described above.

A complete blood count (CBC) and blood chemistry profile were performed before and after the injections of MN-anti-miR10b through the course of the study at the Veterinary Diagnostic Laboratory at Michigan State University.

The study was approved by the Institutional Animal Care and Use Committee of Michigan State University. Written informed consent was obtained from the animal’s owner.

### 2.4 Statistical analysis

Statistical analyses were performed using GraphPad Prism6 Software (version 6, GraphPad software Inc., San Diego, CA, USA). Data are presented as means ± s.d. Statistical comparisons were analyzed using a two-tailed t-test. In all cases, a value of p<0.05 was considered significant.

## 3 Results

### 3.1 miR-10b expression in feline patients with mammary carcinoma (companion cats)

**Figure 1:**
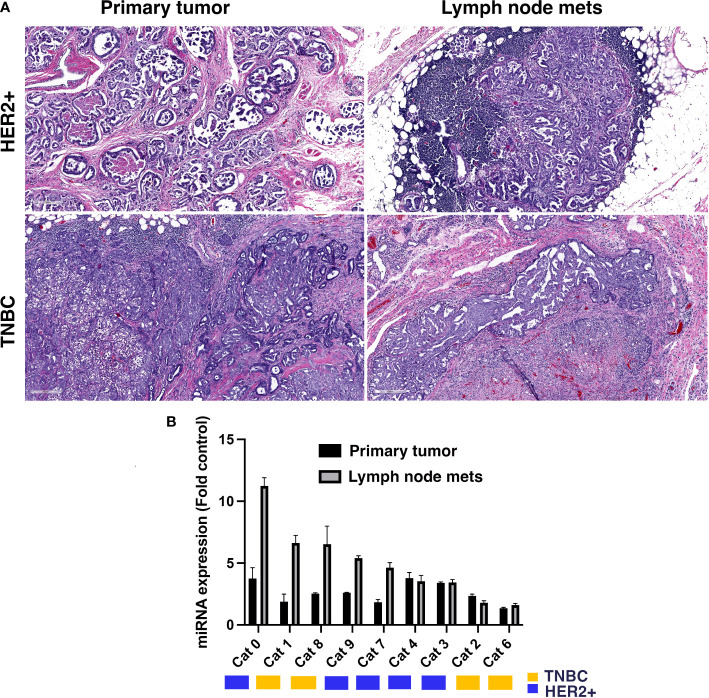
miR-10b expression in primary and metastatic tumors from patients with FMC. **(A)** Representative H&E sections of primary tumors and lymph node metastases from feline patients showing histopathology similar to human breast cancer. Scale bar = 200µm. **(B)** miR-10b expression in primary tumors and lymph node metastases in feline patients. Molecular subtypes are indicated by the color legend. In 55.5% of the tumors miR-10b expression in lymph node metastases was significantly higher than in primary tumors with 60% of them being HER2+ (n = 3, p < 0.05).

To establish the feasibility of targeting miR-10b in FMC we first investigated its expression in primary tumors and metastatic lymph nodes in both HER2+ and TNBC FMCs, which histologically appeared similar to human tumors in terms of architecture and invasive growth patterns (**Figure 1A**). Extensive previous studies in human tumor samples show similar pattern in HER2+ and TNBC primary tumors (24, 25) as well as in HER2+ and TNBC metastatic lesions (26–28). We found that in 55.5% of the tumors, miR-10b expression in lymph node metastases was significantly higher than that in primary tumors with 60% of them being HER2+ (**Figure 1B**). In these tumors miR-10b expression was on average 37.7 ± 7.4% of that in lymph node metastases, which was in agreement with our results in murine metastatic breast cancer models (12).

**Figure 2:**
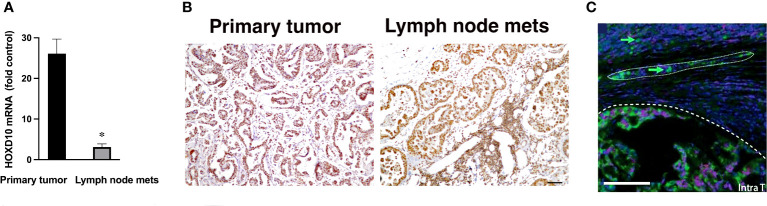
Expression of the direct miR-10b target, HOXD10, in patients with FMC. **(A)** qRT-PCR analysis of HOXD10 expression in primary tumors and lymph node metastases in spontaneous mammary carcinoma in cats. Data from cats 0, 1, 8, 9 and 7 are shown. HOXD10 expression was significantly higher in the primary tumors than in the metastases (n = 3, p < 0.01). Data are represented as mean ± s.d. **(B)** Representative staining for HOXD10, a direct miR-10b target, demonstrating strong signal in the primary tumor (left) and a lower signal in lymph node metastases (right). Scale bar = 100µm. **(C)** Representative ISH field of a primary breast tumor showing miR-10b expression at the invasive front (—) and detached invading cancer cells (arrow) migrating as a group (….) and individually. Scale bar = 100µm.

To further investigate miR-10b expression, we performed qRT-PCR for HOXD10 protein, an established miR-10b direct target, in those paired samples where miR-10b was overexpressed in metastases compared to primary tumors. As shown in **Figure 2A**, the expression of HOXD10 mRNA was significantly lower in lymph node metastases than in primary tumors. Immunohistochemistry for HOXD10 showed that in lymph node metastases the signal was significantly reduced (p<0.05) compared to that in the primary tumor (**Figure 2B** and **Supplementary Figure 1**).

These results are in line with the known pattern of HOXD10 expression, which negatively correlates with miR-10b (9). Similar results have been previously obtained by us in murine models of breast cancer (11). Detailed examination of miR-10b expression in primary tumors by *in situ* hybridization (ISH) demonstrated expression of the miRNA at the highly vascularized invasive fronts of the tumors as well as in detached invading cells (**Figure 2C** and **Supplementary Figure 2**). These results confirmed a similar pattern of miR-10b expression in cats, as has been observed in murine models of human breast cancer as well as in breast cancer patients (9, 29, 30), suggesting that feline mammary carcinoma could be used to model the function of miR-10b in metastatic breast cancer.

### 3.2 Investigation of MN-anti-miR10b in a patient with FMC

To evaluate the potential use and scalability of MN-anti-miR10b for preclinical drug testing we embarked on the first feline patient case study (Cat 0 in **Figure 1**) with HER2+ primary and metastatic tumors (**Supplementary Figure 3A**). The presence of metastatic tumors was confirmed by computed tomography showing defined masses in the abdominal area (**Supplementary Figure 3B**).

In this study two main goals were pursued. First, we wanted to confirm the delivery and accumulation of MN-anti-miR10b in the metastatic lesions. Second, we tested the preliminary tolerability and safety of the preparation. In addition to these two core objectives, we also tested whether MN-anti-miR10b would demonstrate target engagement and inhibition of miR-10b in the metastatic lesions of this animal, as previously observed in murine metastatic breast cancer models (12).

**Figure 3:**
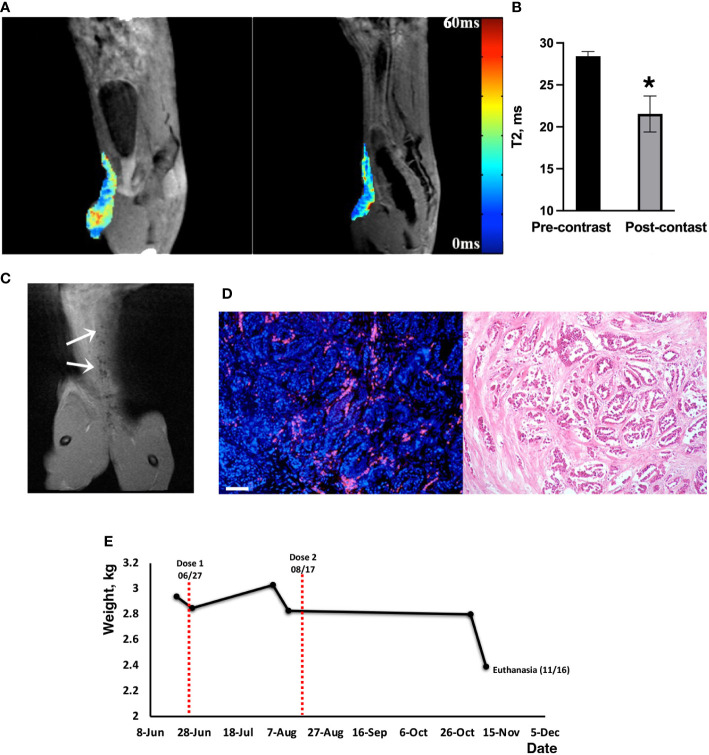
MRI of MN-anti-miR10b delivery to metastatic breast cancer in a feline patient. **(A)** Pre-contrast and post-contrast T2* images (sagittal) of Cat 0 injected with one dose of MN-anti-miR10b. There was a notable loss in signal intensity over the secondary mammary lesion after injection of the therapeutic. **(B)** Quantitative analysis of relaxation times (T2 pre–T2 post, ms) of the tissues, confirming accumulation of MN-anti-miR10b (n=8, p<0.04). Data are represented as mean ± s.d. **(C)** Coronal post-contrast T2-weighted image showing signal voids identified by MN-anti-miR10b (arrows) corresponding to lesions in the abdominal area. **(D)** Left: Fluorescence microscopy showing accumulation of MN-anti-miR10b in the lesion (red – Cy5.5 on the nanoparticle; blue- DAPI); Right: H&E staining of the consecutive slice. Scale bar =100µm. **(E)** Animal weight during the course of the experiment. This symbol * means that the data are statistically significant (i.e., p<0.04).

*In vivo* MRI was used to demonstrate MN-anti-miR10b delivery to metastatic lesions. As shown in **Figures 3A, B**, there was a significant decrease in the T2 relaxation times of the lesions post-injection (28.4 ± 0.5ms pre-contrast vs 21.5 ± 2.1ms post-contrast, p<0.04). Coronal T2-weighted images obtained post injection showed voids of signal intensity characteristic of the accumulation of iron oxide nanoparticles (**Figure 3C**). This observation was confirmed by fluorescence microscopy of a surgical biopsy sample obtained immediately after the imaging session (**Figure 3D** and **Supplementary Figure 4**) showing widespread signal in the Cy5.5 channel after just one injection. Together, these studies provide strong evidence that MN-anti-miR10b, as designed, is delivered to metastatic tumor cells in companion cats after intravenous injection.

Preliminary safety observations showed that the patient tolerated the injections well with no adverse effects such as vomiting, diarrhea, or lethargy. The cat’s vital signs also remained within the normal range and the animal resumed normal activity upon recovery. Complete blood count (CBC) performed before and after the dosing showed no significant difference in values up to 2-3 weeks before euthanasia (**Supplementary Table 1**) when the animal’s health deteriorated due to disease progression. The chemistry profiles were within the normal range except for transient elevation of potassium and Na/K ratio possibly due to dehydration. Liver aspartate transaminase (AST) and creatine kinase (CK) levels were slightly but transiently elevated after injection but returned to normal in two weeks (**Supplementary Table 2**). In some instances, we noticed that parameters that were out of range prior to injection (e.g., chloride and osmolarity calcium) stabilized after dosing. Importantly, though this animal was an older patient (14 years old) and very frail, there was an overall good tolerability and the absence of adverse effects from the injection. Seven weeks after the first dosing, the owner reported good appetite and normal activity levels. Importantly, >5% weight gain (3.03 kg) was recorded (**Figure 3E**). At that time, at the request of the owner and following the recommendation of the management team, a second injection of the therapeutic was administered at the same dose and was tolerated well. CBC, chemistry profiles and animal weight remained unchanged until two weeks prior to euthanasia (**Figure 3E**). While at this point, we cannot establish а direct correlation between this observation and the dosing, we conclude that the therapeutic did not cause weight loss due to toxicity.

**Figure 4:**
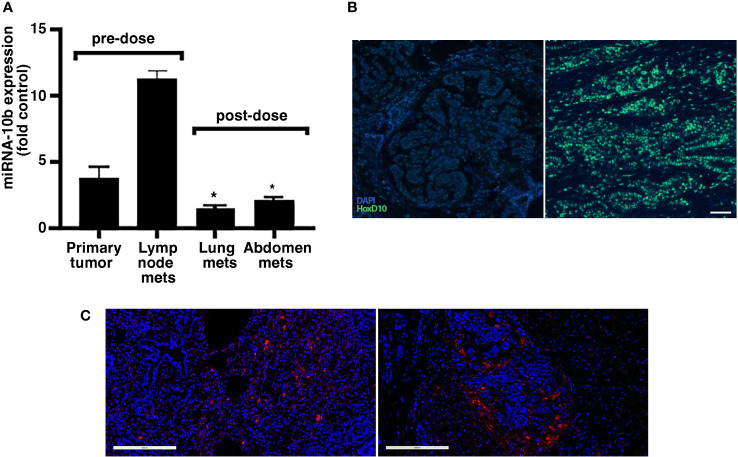
Target engagement and accumulation of MN-anti-miR10b in a patient with FMC. **(A)** qRT-PCR of miR-10b expression in primary tumor and lymph node metastases before dosing, and lung metastases and abdominal metastases three months after second. The expression of miR-10b was significantly reduced post-dosing relative to pre-dosing, indicating successful target engagement (n = 3, p < 0.05). Data are represented as mean ± s.d. **(B)** *In situ* hybridization demonstrating significantly increased HOXD10 expression in metastatic lesions after dosing compared to that in tissues isolated during original tumor excision. Scale bar = 100µm. **(C)** Fluorescence microscopy demonstrating accumulation of MN-anti-miR10b in lung metastases (left) and abdominal area metastases (right) three months after second dose. Scale bar = 200µm. This symbol * means that the data are statistically significant (i.e. p<0.05).

This case of FMC was at an advanced clinical stage. Three months (13 weeks) after the second dose of MN-anti-miR10b, the animal was euthanized due to continuing metastatic growth, renal failure based on urinalysis (high RBC count, not shown), and decreased quality of life. We performed qRT-PCR of multiple metastatic lesions collected at necropsy and found that miR-10b expression was significantly decreased in lung metastases (by ~86.6%) and metastases in the abdominal area (by ~81.6%) compared to that in the lymph node metastases removed during the original surgery (**Figure 4A**). Importantly, expression of the miR-10b target HOXD10 was significantly increased in metastatic lesions compared to that in metastatic lymph nodes isolated during surgery prior to animal enrollment in the study (p<0.01; **Figure 4B** and **Supplementary Figure 5**). The continued presence of MN-anti-miR10b in the metastatic lesions was observed microscopically (**Figure 4C**, left and right and **Supplementary Figure 6**) even at three months post-dosing, reflecting the relatively low rate of cell division of spontaneous malignancies and is consistent with the fact that decreases in the intracellular concentration of MN-based agents are dominated by loss due to cell division but not exocytosis (12). No Cy5.5-derived fluorescence was observed in tissues obtained prior to dosing, confirming that the post-dosing signal indeed reflected the presence of MN-anti-miR10b (**Supplementary Figure 7**).

Taken together, these results suggest that in spontaneous models of metastatic breast cancer, such as feline mammary carcinoma, MN-anti-miR10b has an acceptable safety and tolerability profile, demonstrates a tissue distribution that favors lasting delivery to metastatic lesions, and exhibits effective target engagement in metastatic tumor cells.

## 4 Discussion

Previously, we have shown that miR-10b plays a pivotal role in supporting metastatic cell viability and proliferation (11, 12). To inhibit miR-10b, we designed and tested an miR-10b-specific therapeutic, which caused lasting regression of established metastases in immunocompromised (11, 12) and immunocompetent murine models (13). This report serves as the logical next step towards the clinical development of MN-anti-miR10b and is the first in a series of studies aimed at investigating the applicability of feline mammary carcinoma, a spontaneous cancer, as a translational model, bridging human clinical trials centered on noncoding RNAs as therapeutic targets.

Investigation of miR-10b expression in feline tissues confirmed the diversity and heterogeneity of FMC presentation in terms of miR-10b expression and tumor receptor positivity, which was similar to that in humans. This points to the necessity to obtain evidence of miR-10b expression from biopsy samples to stratify patients who can benefit from this therapy. In human cancer, miR-10b expression is significantly increased in later stage patients (31–34) and in those with more aggressive types (35). In future clinical trials it is reasonable to expect that in order to guide treatment, patients will be selected based on their levels of miR-10b expression, similar to the current standard diagnostic tests with other cancer markers, such as HER2+ (36, 37).

To translate our earlier successful studies in mice to humans, investigating the effectiveness of the therapeutic in relevant spontaneous diseases in large animals is necessary. The case study with our first-in-class miRNA targeted therapeutic presented here demonstrated its delivery to metastatic lesions using MRI, which is an important step in preclinical development of our approach. Initial safety studies demonstrated good tolerability and the general lack of toxicity of the therapeutic, which serves as another important milestone in its translation. Furthermore, we obtained proof of target engagement by MN-anti-miR10b, manifested as a significant decrease in miR-10b expression after two injections 7 weeks apart. It is important to note that efficacy studies were not part of this investigation, and the dose of the therapeutic used here was lower than the animal equivalent dose (AED) calculated based on the effective dose determined in our previous rodent studies (11–13). However, even at this reduced dose and suboptimal schedule, we achieved a significant inhibition of the miR-10b target with no toxicity. Although euthanasia was performed due to disease progression and deteriorated health, the patient survived for five additional months compared to the animal’s life expectancy prior to dosing.

Our future studies will include a clinical trial in companion animals with both TNBC and HER2+ molecular subtypes to investigate the safety and efficacy of MN-anti-miR10b, potentially in combination with conventional chemotherapy. Importantly, imaging will play a significant role in the assessment of drug delivery, as shown here. Notwithstanding the need for additional therapeutic and toxicology studies, our findings establish the feasibility and suggest the robustness and safety of a novel first-in-class therapeutic approach against metastatic breast cancer that could ultimately improve clinical outcomes in both feline and human patients.

## Data availability statement

The original contributions presented in the study are included in the article/**Supplementary Material**. Further inquiries can be directed to the corresponding author.

## Ethics statement

The animal study was reviewed and approved by Institutional Animal Care and Use Committee of Michigan State University. Written informed consent was obtained from the owners for the participation of their animals in this study.

## Author contributions

Conceptualization, AM and VY-G; Methodology, NS, PS, VY-G, PW, BY, MK, and LS; Software, PW; Validation, NS, AH, AM, and BY; Investigation, AM, ZM, LS, and VY-G; Resources, AM, VY-G, PS, and ZM; Data Curation, AM, VY-G, LS, and ZM; Writing – Original Draft Preparation, NS and AM; Writing – Review & Editing, NS, PS, AH, VY-G, PW, MK, LS, ZM, and AM; Visualization, AM; Supervision, AM; Project Administration, AM and VY-G; Funding Acquisition, AM and LS. All authors contributed to the article and approved the submitted version.

## Funding

This work was supported in part by R21CA226579 and R01CA258314 to LS and 1R01CA261691 to AM.

## Acknowledgments

The authors would like to thank Drs. Erik Shapiro and Christiane Mallett for their valuable help with the MR imaging.

## Conflict of interest

ZM and AM are scientific founders and shareholders at TransCode Therapeutics Inc.

The remaining authors declare that the research was conducted in the absence of any commercial or financial relationships that could be construed as a potential conflict of interest.

## Publisher’s note

All claims expressed in this article are solely those of the authors and do not necessarily represent those of their affiliated organizations, or those of the publisher, the editors and the reviewers. Any product that may be evaluated in this article, or claim that may be made by its manufacturer, is not guaranteed or endorsed by the publisher.
