# Supplementary material for: Case report: MicroRNA-10b as a therapeutic target in feline metastatic mammary carcinoma and its implications for human clinical trials
Source: Front Oncol. 2022 Oct 26;12:959630. doi: 10.3389/fonc.2022.959630 (PMC9643803; doi:10.3389/fonc.2022.959630)
Supplement: Supplementary file 1 [file DataSheet_1.pdf]

Supplementary Material

1 Supplementary Figures and Tables

1.1 Supplementary Figures

Figure

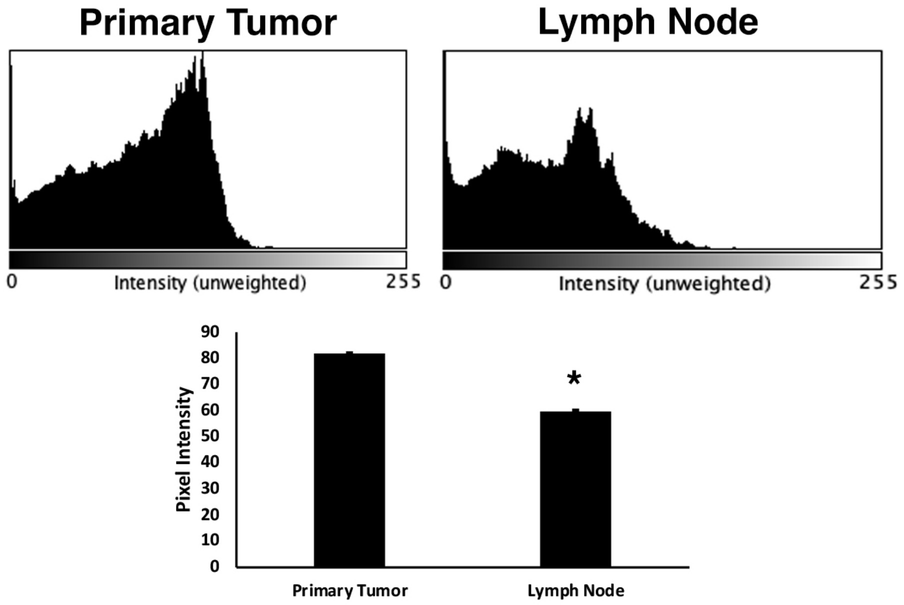

Supplementary Figure 1. Histogram analysis (left) and quantitation (right) of pixel-by-pixel signal intensity from the primary tumor and metastatic lymph nodes in Fig. 2B showing a significant difference in HOXD10 staining ($p<0.05$).

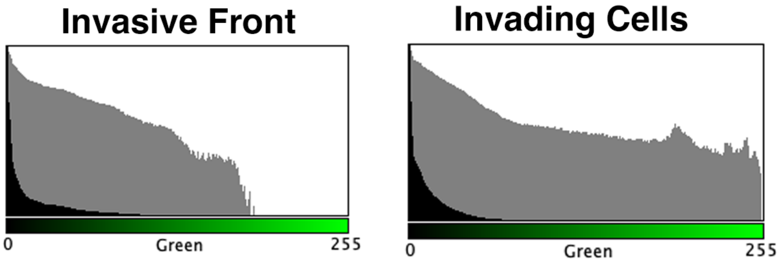

Supplementary Figure 2. Histogram analysis (log scale) of pixel-by-pixel signal intensity corresponding to cells stained for miRNA-10b in Fig. 2C, showing positivity for miRNA-10b expression in both cell populations.

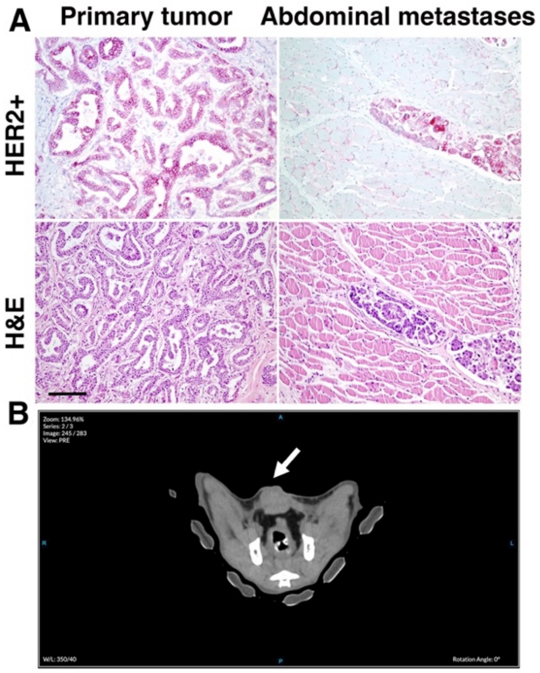

Supplementary Figure 3. A) Case 0 patient's primary (left) and metastatic (right) tumors stained for HER2+ (top row) and H&E (bottom row). Scale bar=200µm. B) CT imaging showing metastatic tumors in the abdominal area of the patient with FMC (arrow).

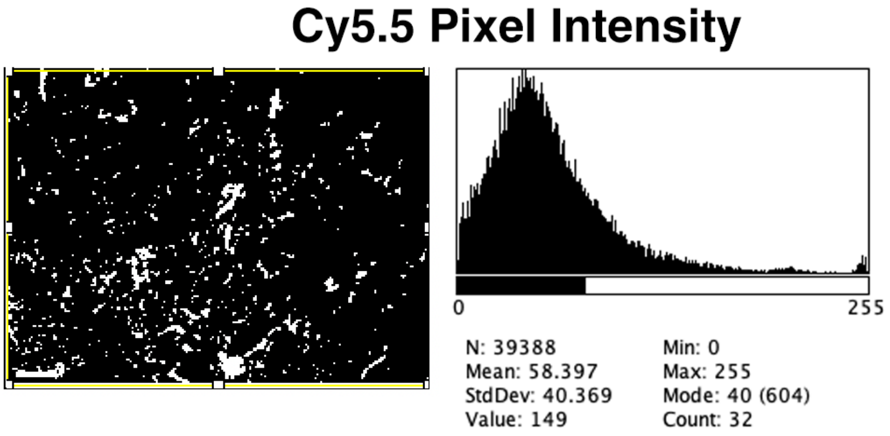

Supplementary Figure 4. Processed image (left) and corresponding histogram (right) of Cy5.5 fluorescence intensity in biopsy samples (Fig. 3D) obtained 24 hours after dosing, indicating the presence of the therapeutic in the tissue.

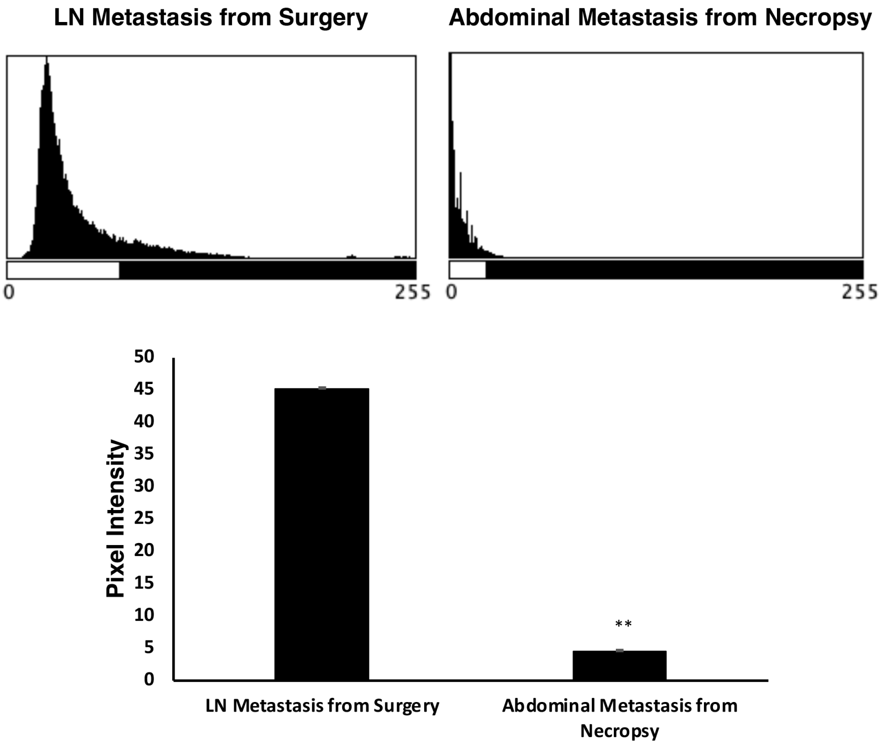

Supplementary Figure 5. Histogram analysis (top) and quantitation (bottom) of pixel-by-pixel signal intensity (Fig. 4B) in metastatic tissue obtained during original surgery and at necropsy after dosing showing significant difference in HOXD10 expression ($p<0.01$).

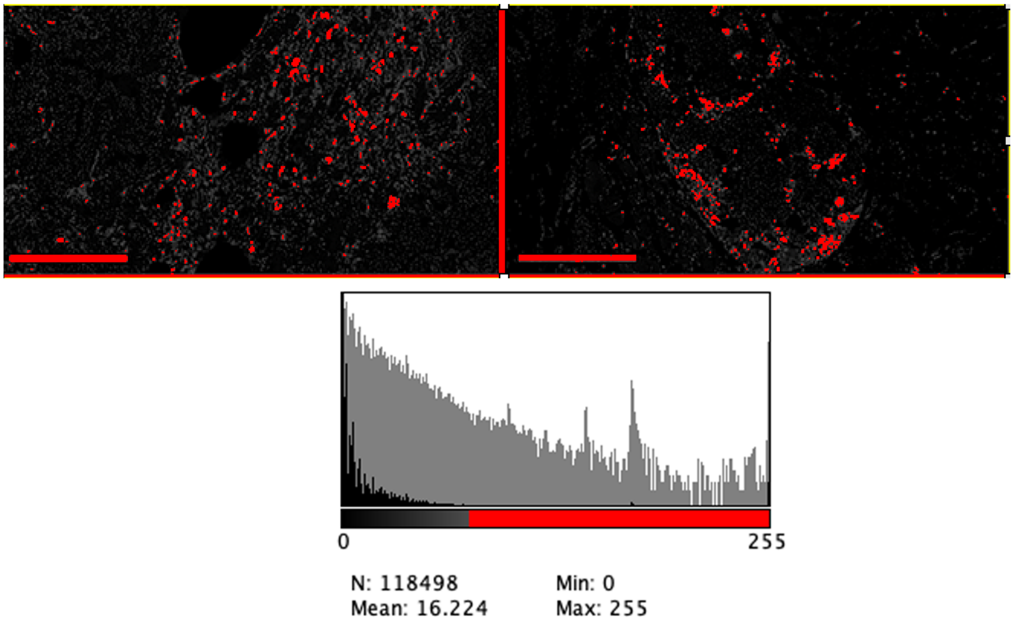

Supplementary Figure 6. Processed color-coded image (top) and corresponding histogram (log scale, bottom) of Cy5.5 fluorescence intensity in necropsy samples from lung (left) and abdominal (right) metastasis (Fig. 4C) indicating the presence of the therapeutic at three months after dosing.

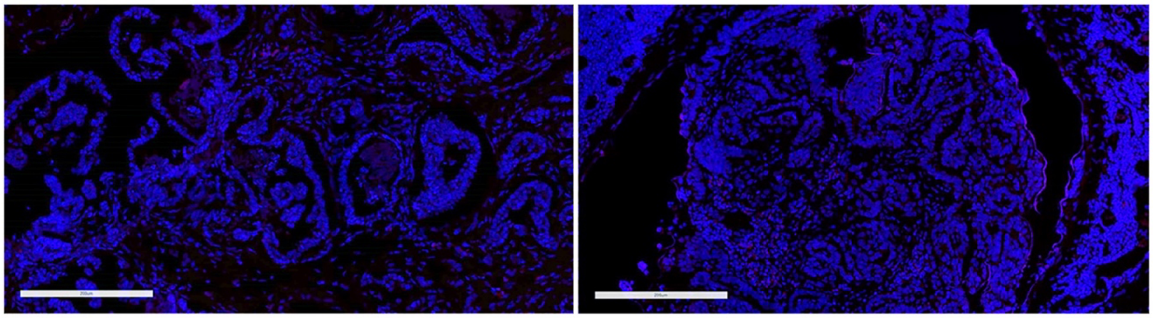

Supplementary Figure 7. Tissues from the primary tumor (left) and metastatic lymph nodes (right) obtained during the original surgery did not show Cy5.5 signal from the therapeutic. DAPI staining of nuclei (blue). Scale bar = 200µm.

1.2. Supplementary Tables

Table 1. Complete blood count.

1st dose (6/27) → 6/27; 2d dose (8/17) → between 8/3 and 9/28; Euthanasia (11/16) → after 10/11

CBC	6/20	6/27	6/28	7/13	7/19	8/3	9/28	10/9/	10/11	Ref Range	Units
RBC	**6.7 L**	**7.0 L**	**4.3 L**	**6.8 L**	**6.4 L**	**6.6 L**	**6.8 L**	**6.5 L**	**4.9 L**	[7.9-11.6]	x10.e6/uL
Hgb	**10.3 L**	**10.6 L**	**6.5 L**	**10.6 L**	**9.9 L**	**10.7 L**	**10.2 L**	**9.9 L**	**7.5 L**	[11.9-17.5]	g/dL
Hct	**32 L**	34	**20 L**	**33 L**	**31 L**	**31 L**	**32 L**	**31 L**	**23 L**	[34-51]	%
MCV	49	48	47	48	48	47	48	48	47	[38-52]	fL
MCH	15	15	15	16	16	16	15	15	15	[13-17]	pg
MCHC	**32 L**	**31 L**	**32 L**	**32 L**	**32 L**	34	**32 L**	**32 L**	**32 L**	[33-37]	g/dL
CHCM	**30 L**	**30 L**	**30 L**	**31 L**	32	32	32	**31 L**	32	[32-35]	g/dL
RDW	16	**17 H**	16	**17 H**	**18 H#**	16	**17 H**	**17 H**	**17 H**	[14-16]	%
Platelet	249	215	**174 L**	389	427	243	252	235	263	[179-569]	x10.e3/uL
MPV	17.5	**18.9 H**	17.8	**21.1 H**	**19.7 H**	17.8	**18.2 H**	**18.7 H**	**19.5 H**	[9.8-18.1]	fL
WBC	**29.5 H**	**16.8 H#**	**16.8 H**	**25.2 H**	**18.8 H**	**30.3 H**	**35.6 H**	**20.8 H**	**24.4 H**	[4.4-15.6]	x10.e3/uL
Neut #	**25.1 H**	**14.6 H**	**14.3 H**	**24.7 H**	**17.5 H**	**27.3 H**	**34.0 H**	**19.9 H**	**22.6 H**	[2.3-11.8]	x10.e3/uL
Lymphnocyte #	0.6	1	1	0.5	0.8	0.9	0.7	**0.6 L**	1	[0.7-6.3]	x10.e3/uL
Monocyte #	0.3	0.2	0.2	0	0	0	**0.5 H**	0.2	0.2	[0.1-0.4]	x10.e3/uL
Eosinophil #	**3.2 H**	0.8	1.2	0	0.6	**1.8 H**	0.4	0.2	0.5	[0.2-1.3]	x10.e3/uL
Neut Pct	85	87	85.3	98	93	90	95.5	95.7	92.8		%
Lymphnocyte Pct	2	6	5.7	2	4	3	2	2.7	4.1		%
Monocyte Pct	1	1	1.5	0	0	0	1.3	0.8	0.9		%
Eosinophil Pct	11	5	7.4	0	3	6	1.1	0.7	2.1		%

Manual count; H- high; L-low.

Table 2. Blood chemistry. 1st dose → 6/27; 2d dose (8/17) → 8/29; Euthanasia → 10/11

**Clinical Chemistry**	**6/20**	**6/27**	**6/28**	**7/13**	**7/19**	**8/3**	**8/29**	**9/21**	**9/10**	**10/11**	**Ref Range**	**Units**
Urea Nitrogen	29	28	24	23	**41 H**	36	**45 H**	**37 H**	**42 H**	34	[19-36]	mg/dL
Creatinine	1.2	1.2	1	1.1	1.6	1.5	1.6	1.5	1.6	1.2	[1.0-2.3]	mg/dL
Sodium	154	155	151	150	152	152	151	155	150	155	[145-155]	mmol/L
Potassium	4.4	4.2	**3.4 L**	3.9	4.7	4	4.6	3.9	4.3	**3.7 L**	[3.8-5.4]	mmol/L
Chloride	**124 H**	123	121	121	119	119	118	121	120	123	[110-123]	mmol/L
TCO2	18	16	22	16	18	21	18	16	16	19	[13-22]	mmol/L
Na/K Ratio	35	37	**44 H**	38	32	38	33	40	35	**42 H**	[28-40]	
Anion Gap	16	20	**11 L**	17	20	16	20	22	18	17	[15-28]	mmol/L
Osmolarity Calc	**326 H**	**325 H**	314	315	323	322	322	**328 H**	321	**326 H**	[302-323]	mOs/L
Calcium	**8.8 L**	9.2	**8.5 L**	**8.5 L**	9.1	9.1	9.2	9.4	9.1	9.1	[9.1-10.7]	mg/dL
Phosphorus	3.6	3.6	5.7	3	5.6	3.3	5	4.3	5.3	4.4	[2.7-5.7]	mg/dL
Magnesium	1.9	2	1.9	**1.7 L**	2.1	1.9	2.2	2	2	**1.6 L**	[1.8-2.5]	mg/dL
Iron	62	55	129	**34 L**	83	90	89	65	103	**226 H**	[45-157]	ug/dL
Total Protein	**6.2 L**	6.8	**5.5 L**	7	7	6.7	6.6	6.8	6.5	**6.0 L**	[6.5-7.8]	g/dL
Albumin	**2.4 L**	**2.6 L**	**2.1 L**	**2.5 L**	**2.3 L**	**2.5 L**	**2.5 L**	**2.6 L**	**2.6 L**	**2.3 L**	[3.0-3.9]	g/dL
Globulin Calc	3.8	4.2	3.4	4.5	4.7	4.2	4.1	4.2	3.9	3.7	[2.8-4.7]	g/dL
Glucose	129	90	**56 L**	115	84	100	**69 L**	86	103	**68 L**	[78-143]	mg/dL
Amylase	951	899	767	844	1002	993	963	983	1056	897	[476-1,779]	U/L
Total Bili	0.1	0.1	0.1	0.1	0.1	0.1	0.1	0.1	0.1	0.1	[0.1-0.3]	mg/dL
Direct Bili	0	0	0	0	0	0	0	0	0	0	[0.0-0.1]	mg/dL
Indirect Bili	0.1	0.1	0.1	0.1	0.1	0.1	0.1	0.1	0.1	0.1	[0.1-0.3]	mg/dL
ALP	30	40	33	30	26	30	32	27	34	32	[13-48]	U/L
ALT	40	43	39	33	37	38	36	26	30	33	[25-76]	U/L
AST	**39 H**	36	**41 H**	28	33	**39 H**	**43 H**	**44 H**	**37 H**	**41 H**	[14-36]	U/L
CK	231	267	**532 H**	81	99	180	167	188	135	154	[46-490]	U/L
Chol	155	172	124	144	190	185	186	234	186	158	[72-248]	mg/dL

H- high; L-low
